# Is It Possible to Predict the Future in First-Episode Psychosis?

**DOI:** 10.3389/fpsyt.2018.00580

**Published:** 2018-11-13

**Authors:** Jaana Suvisaari, Outi Mantere, Jaakko Keinänen, Teemu Mäntylä, Eva Rikandi, Maija Lindgren, Tuula Kieseppä, Tuukka T. Raij

**Affiliations:** ^1^Mental Health Unit, National Institute for Health and Welfare, Helsinki, Finland; ^2^Department of Psychiatry, McGill University, Montreal, QC, Canada; ^3^Bipolar Disorders Clinic, Douglas Mental Health University Institute, Montreal, QC, Canada; ^4^Department of Psychiatry, Faculty of Medicine, University of Helsinki, Helsinki, Finland; ^5^Department of Neuroscience and Biomedical Engineering, and Advanced Magnetic Imaging Center, Aalto NeuroImaging, Aalto University School of Science, Espoo, Finland; ^6^Department of Psychology and Logopedics, University of Helsinki, Helsinki, Finland

**Keywords:** first-episode psychosis, remission, recovery, comorbidities, mortality, prediction

## Abstract

The outcome of first-episode psychosis (FEP) is highly variable, ranging from early sustained recovery to antipsychotic treatment resistance from the onset of illness. For clinicians, a possibility to predict patient outcomes would be highly valuable for the selection of antipsychotic treatment and in tailoring psychosocial treatments and psychoeducation. This selective review summarizes current knowledge of prognostic markers in FEP. We sought potential outcome predictors from clinical and sociodemographic factors, cognition, brain imaging, genetics, and blood-based biomarkers, and we considered different outcomes, like remission, recovery, physical comorbidities, and suicide risk. Based on the review, it is currently possible to predict the future for FEP patients to some extent. Some clinical features—like the longer duration of untreated psychosis (DUP), poor premorbid adjustment, the insidious mode of onset, the greater severity of negative symptoms, comorbid substance use disorders (SUDs), a history of suicide attempts and suicidal ideation and having non-affective psychosis—are associated with a worse outcome. Of the social and demographic factors, male gender, social disadvantage, neighborhood deprivation, dysfunctional family environment, and ethnicity may be relevant. Treatment non-adherence is a substantial risk factor for relapse, but a small minority of patients with acute onset of FEP and early remission may benefit from antipsychotic discontinuation. Cognitive functioning is associated with functional outcomes. Brain imaging currently has limited utility as an outcome predictor, but this may change with methodological advancements. Polygenic risk scores (PRSs) might be useful as one component of a predictive tool, and pharmacogenetic testing is already available and valuable for patients who have problems in treatment response or with side effects. Most blood-based biomarkers need further validation. None of the currently available predictive markers has adequate sensitivity or specificity used alone. However, personalized treatment of FEP will need predictive tools. We discuss some methodologies, such as machine learning (ML), and tools that could lead to the improved prediction and clinical utility of different prognostic markers in FEP. Combination of different markers in ML models with a user friendly interface, or novel findings from e.g., molecular genetics or neuroimaging, may result in computer-assisted clinical applications in the near future.

## Introduction

Naturalistic follow-up studies have found highly divergent outcomes in first-episode psychosis (FEP) (1, 2). While an episodic course is the most common (1) and the majority of patients with FEP initially achieve remission (2), a minority experience early sustained recovery (3), or have an antipsychotic treatment-resistant illness from the onset of the illness (4). The challenge for the clinician treating patients with FEP is how to predict these different disease trajectories and make the best treatment choices for individual patients.

A growing concern in recent years has been the multiple physical comorbidities in people with schizophrenia and other psychotic disorders (5) and the premature mortality caused by these comorbidities (6, 7). Antipsychotic medication contributes to these problems by causing weight gain, impaired glucose tolerance and dyslipidemias. However, antipsychotics differ in their propensity to cause these side effects (8), and there is also considerable individual variation in the sensitivity to these side-effects. Moreover, other factors, possibly even shared etiological mechanisms, contribute to the development of comorbidities like diabetes (9). The personalized treatment of FEP would benefit from biomarkers identifying the patients at greatest risk for medication side effects and comorbidities.

While cardiovascular and pulmonary diseases are overall the most important causes of premature mortality (6), in the first years of illness increased mortality is mainly caused by suicide (10). Suicide prevention is one of the key goals in the treatment of FEP (11), and yet another important outcome for which the clinician needs to identify relevant risk factors.

In addition to these main areas where outcome prediction is needed—remission, recovery, physical comorbidities, and suicide risk—some domains in the psychosis phenotype, for example cognitive functioning, can be considered both as predictors of the long-term course and as relevant long-term outcomes.

This selective review aims to provide a synthesis of the current literature on outcome prediction for FEP. We also discuss some methodologies and tools that could enhance possibilities to predict the future in FEP.

## Outcome prediction: current evidence

### Clinical and sociodemographic factors

Remission in FEP refers to symptomatic remission; the Remission in Schizophrenia Working Group defined it as maintaining a symptom level of mild or less regarding positive, negative, and disorganized symptoms over a 6-month period (12). Recovery is a broad concept that should take both clinical symptoms and psychosocial functioning into account, with subjective recovery being an important component (13). While the Remission in Schizophrenia Working Group criteria for remission have achieved gold standard status in research, no uniform criteria exist for recovery. In a recent meta-analysis of longitudinal FEP studies, the pooled proportion of patients achieving remission after an average of 5.5 years follow-up was 58%, and studies conducted in more recent years found higher remission rates (2). However, the proportion achieving recovery after an average of 7.2 years follow-up was only 38%, and this was lower in both more recent studies and in studies with longer follow-up times (2). After 2 years of follow-up, the proportion achieving recovery was stable, suggesting that the poor outcome trajectory is already apparent during the early stages of illness (2). Recovery rates were also lower in studies requiring a longer duration of good functioning and the absence of symptoms (2). Schizophrenia was associated with lower remission and recovery rates than other psychotic disorders (2).

Clinical features related to the first psychotic episode were surprisingly poor predictors of remission and recovery in the meta-analysis by Lally et al. (2). Remission status was not predicted by the severity of psychotic symptoms at baseline, the duration of untreated psychosis (DUP), treatment adherence, employment status, or marital status (2). In another meta-analysis focusing on relapse risk following the first psychotic episode, significant risk factors were medication non-adherence, persistent substance use disorder (SUD), the carer's critical comments and poor premorbid adjustment (14). In the Etiology and Ethnicity in Schizophrenia and Other Psychoses (AESOP) study, patients with an initial diagnosis of non-affective psychosis, patients living in a deprived area, and male patients had a poorer 10-year outcome than other patients (1). Some ethnic minorities had a worse outcome, which was partly explained by social disadvantage (15). In another large longitudinal FEP study, deterioration in premorbid social functioning, DUP of ≥26 weeks, a core schizophrenia spectrum disorder and no remission within the first 3 months all predicted a longer time in psychosis during a 10-year follow-up (16). Regarding shorter-term outcomes, unemployment, poor education, functional deficits, unmet psychosocial needs, previous depressive episodes, male sex, and suicidality predicted poor 1-year outcomes in a machine learning (ML) study that utilized data from the European First Episode Schizophrenia Trial (17).

Recent studies have reported inconsistent findings regarding the effects of the discontinuation of antipsychotic treatment after achieving remission of FEP. A meta-analysis of treatment discontinuation vs. maintenance treatment strategies in FEP clearly showed that the relapse risk is higher in the discontinuation group (18). In a recent register-based follow-up study, the discontinuation of antipsychotic medication was a strong predictor of both rehospitalization and premature mortality even after several years of continuous outpatient antipsychotic treatment (19). While one study found that early dose reduction or the discontinuation of antipsychotic treatment following a 6-month remission were both associated with a better long-term outcome (20), another recent randomized clinical trial (RCT) found that discontinuation after 1 year of antipsychotic maintenance treatment was associated with a poorer 10-year clinical outcome (21). However, in the AESOP study, 12.5% of FEP patients had early sustained recovery with no relapses over a 10-year follow-up period, and their median duration of antipsychotic treatment was only 53 days (3). Predictors of early sustained remission were female gender, being employed, being in a relationship, having have a short DUP, and having mania or a brief psychotic disorder diagnosis (3). Unfortunately, all these predictors are relatively common and not specific enough to evaluate who would possibly benefit from antipsychotic discontinuation after FEP.

About a quarter of FEP patients are treatment-resistant, that is, they show little or no improvement in psychotic symptoms after two consecutive treatments with different antipsychotics of adequate dose and duration (4, 22). The majority of treatment-resistant patients are treatment resistant from the onset of illness (4, 22). In two large FEP studies, treatment resistance was predicted by a younger age at onset, a schizophrenia diagnosis, negative symptoms, and a longer DUP (4, 22).

### Insight and resilience

Insight has been of special interest as a predictor of the outcome of FEP, as defects in insight may possibly arise from the same functional and structural brain pathology as psychosis itself (23, 24). Cognitive insight at baseline, including measures of both self-reflectiveness and self-certainty, has been shown to predict overall psychopathology at 1-year follow-up (25). However, after a 4–year follow-up, only the self-reflectiveness subscale was associated with symptom remission (26). In FEP, cognitive insight has been associated with cortical thickness (27), and the self-certainty subscale has been associated with changes in a frontal network (28). However, greater insight has also predicted suicidality after FEP (29).

Impaired clinical insight, which is somewhat separable from cognitive insight, has been associated with poorer social functioning, more re-hospitalizations and treatment non-adherence (30). In one study, the best predictors of relapse within 2 years after FEP were cannabis use before relapse and poor insight (insight being measured at a 2-month follow-up) (31). Poor insight may prolong the DUP (32) and predict non-adherence to medication treatment in FEP patients (33), although the results in prospective studies with FEP samples are somewhat mixed with regards to treatment adherence (34). Interestingly, baseline self-rated insight and objective insight at 6 weeks predicted hospital readmission in a sample consisting mostly of first-episode non-affective psychosis patients, whereas baseline objective insight and self-rated insight at 6 weeks were not significant predictors (35). Clinical insight changes over time in FEP, and likewise its correlation with symptoms and psychosocial functioning is not consistent in the early course of illness and the later course of illness (36). In a 3-year follow-up of a large FEP cohort, improvement in insight in the early course of illness was associated with increasing depressive symptoms, but this association disappeared later (36). Improving insight was associated with improving psychosocial functioning in the early course of the illness, but later the relationship became more complex (36). This reflects a complex social identity process that occurs after a first psychotic episode, in which insight is more than just a simple trait or state feature (36).

Resilience, a personality trait manifesting in a response to adversity, also plays a role in recovery, as it implies successful adaptation despite difficult experiences. Resilience is linked to psychological well-being or positive mental health, which is increasingly seen as an important treatment target on its own and which tends to be at a low level, particularly for patients with active delusions (37). Fully recovered patients with first episode schizophrenia—defined as patients living independently, working or studying, having absent or stably mild symptoms for 2 years, and having social contacts and participation—showed a significant increase in resilience at 4-year follow-up (20, 38). These results indicate that individual differences in resilience will differently affect the recovery process (20), stressing the importance of taking resilience into account in outcome studies and using resilience-building strategies. Measures of resilience and psychological well-being might be important as outcome predictors, but currently they have rarely been studied in large FEP cohorts.

### Cognition

Cognitive deficits are common in FEP throughout all phases of the illness, including impairment in working memory, processing speed, verbal and visual learning, reasoning, and social cognition. Cognitive deficits are already present during the prodromal phases of the illness (39) and are not correlated with the DUP (40). Although it is widely believed that symptom fluctuation usually does not affect cognitive performance (41), an association between the level of negative symptoms and cognitive deficits has been reported several times, with cognitive performance improving when negative symptoms ease off (42).

There does not seem to be a cognitive decline in the first years of illness (39), a possible exception being progressive verbal memory deterioration (42). However, with longer follow-up times, cognitive functioning may continue to decline following the first episode. Over a lifespan, periods of cognitive deterioration in schizophrenia appear to be a period before the first episode and another at approximately 65 years of age (43). In the long term, people with schizophrenia are also at increased risk of dementia (11). Dementia is a very long-term outcome and not very relevant for the treatment of FEP. Recent research suggests that the increased risk of dementia may be mediated through comorbidities like cardiovascular disease (CHD) (44), whereas there is no genetic correlation between schizophrenia and Alzheimer's disease (45).

Cognitive functioning at the beginning of the psychotic illness may predict the illness course and functional outcome such as self-care, work performance and social functioning (46). Remission and relapses of FEP within the first 2 years of illness may be predicted by verbal fluency, memory and social cognition, and persistent negative symptoms and functional outcomes may be predicted by verbal memory (47). Social cognition has been found to specifically predict everyday community functioning, such as independent living skills, and social and work functioning (48). Of the social cognitive domains, the theory of mind might be an especially important treatment target due to its associations with functional outcome (48). In one study, FEP patients with preserved intelligence quotient (IQ) at psychosis onset had better outcomes at 3 years than other patients in terms of disorganization and negative symptoms, index admissions, and occupational outcome (49). Severe cognitive impairment by the time of the first psychotic episode may thus predict a more severe illness. Deficits in cognitive functions may affect adherence, insight, social skills, and one's overall capacity to take care of oneself thus, leading to a worse symptomatic and functional outcome. On the other hand, premorbid adjustment, motivation, negative symptoms, and insight may moderate the impact of cognition on these functional outcomes (46).

The functional outcome of FEP has also been predicted with premorbid cognitive reserve (50, 51). Higher premorbid IQ and educational attainment may help a person to cope with the effects of the disease, thus affecting the neuropsychological, functional and clinical outcome of FEP. High cognitive reserve may help the individual use compensatory abilities and is associated with better insight (49).

### Brain imaging

Brain imaging methods have been used to differentiate patients from healthy controls and to predict the long-term outcome of FEP. In recent years, several studies have used either structural or functional brain imaging, sometimes together with clinical information and other biomarker information, to classify FEP patients or patients with clinical high-risk symptoms from healthy control subjects (52–58). Accuracies in these ML studies that use one imaging modality have ranged from 66% (52, 55) to 87% (53), and the combination of structural magnetic resonance imaging (MRI) and diffusion tensor imaging data has been reported to result in enhanced accuracy: 93% (54). These classification studies have built the grounds upon which to study whether FEP patients can be further classified into subgroups with different outcomes.

Early multivariate ML studies suggested that continuous and remitting courses of illness were predictable based on structural MRIs alone, with accuracies of 58% (59) and 70% (60). However, these findings were not replicated in other research centers nor in the data pooled across centers (60). In a follow-up study by Pina-Camacho and co-workers, brain volumetric measures did not enhance the classification accuracy of schizophrenia spectrum vs. other psychoses beyond the 81% classification accuracy achieved using clinical symptoms alone (61).

Most earlier brain imaging studies on outcome prediction have used univariate methods. Univariate findings which may predict outcomes include alterations in rhythmic activity in electroencephalogram (EEG) (62); a prefrontal MRI spectroscopic marker of neuronal integrity (63); striatal dopamine-2 receptor binding potential (64); the integrity of the frontotemporal white matter tracts (65); abnormal gyrification of the cerebral cortex (66, 67); white matter network organization (68); and the volumes of the ventricles (69) and the temporal lobe in general (70), and the volumes of the hippocampus (71, 72) and the superior temporal gyrus (73) in particular. For a review on structural MRI measures as predictors of outcome, see Dazzan et al. (74). Outcome prediction based on any of the univariate findings is too inaccurate to be clinically useful (75). An exception might turn out to be using a lack of elevated dopamine synthesis capacity, which has been associated with antipsychotic treatment resistance in two studies (76, 77). These findings, however, provide important information for more complex ML models with potential for clinically sufficient prediction accuracy.

### Genetics

The etiological significance of genetic factors in psychotic disorders is substantial: the heritability of schizophrenia spectrum and bipolar disorders is around 65–85% (78–80). Numerous studies have correlated variants in schizophrenia candidate genes with phenotypic features, sometimes also with outcome measures. However, recent genetic studies have questioned the validity of previously suggested schizophrenia candidate genes (81, 82). Therefore, we focus here on the potential value of genome-wise significant findings from genome-wide association (GWA) studies and rare damaging variants identified from GWA or exome/whole genome sequencing studies in predicting the outcomes of psychosis.

The number of identified, genome-wide significant genetic loci associated with schizophrenia in GWA studies currently increases in proportion to sample size, being already over 100 in 2014 (83) and 145 in the most recently reported GWA study (84). Consequently, polygenic risk score (PRS) estimates derived from GWA studies have become increasingly accurate. They have been used to predict both treatment response and long-term outcome. A higher schizophrenia PRS has been associated with worse treatment response (85), a higher likelihood of being in clozapine treatment (86), more frequent hospital admissions (87), and more severe negative symptoms (88) in patients with schizophrenia. One fairly large study, however, failed to find an association between PRS and poor treatment response in schizophrenia (89). In patients with bipolar disorder, a high schizophrenia PRS is associated with an increased risk of having psychotic symptoms (88), particularly mood-incongruent psychotic symptoms (90). In a FEP study sample, the schizophrenia PRS was predictive of a future schizophrenia diagnosis (as opposed to the diagnosis of other psychotic disorders), although its discriminatory accuracy was relatively modest (91). The bipolar disorder PRS, in turn, is associated with having more severe manic symptoms in patients with schizophrenia, but also with psychotic symptoms in patients with bipolar disorder, and a PRS calculated from the variants shared between bipolar disorder and schizophrenia is associated with psychotic symptoms in bipolar disorder and more severe negative symptoms in schizophrenia (88). The schizophrenia PRS has been associated with lower hippocampal volume in FEP patients (92), but overall the associations between the schizophrenia PRS and psychosis endophenotypes are modest (93). The benefit of a PRS is that it is a stable trait feature. It could be a useful component of larger predictive algorithms in the future, but this still needs more research.

Besides the polygenic background of common variants which individually have a very small effect, rare variants have been identified which are present in a very small proportion of the population but have a substantially larger effect on schizophrenia/psychosis risk. The Psychiatric Genomics Consortium recently validated six deletions and two duplications of significant risk factors for schizophrenia, and identified several novel ones (94). However, while these copy number variants (CNVs) are associated with an up to 60-fold elevated risk of schizophrenia in case-control studies (94), general population-based studies have also identified people carrying the same CNVs who have normal functioning and only minimal problems in cognitive tests (95). In exome and whole-genome sequencing studies, the first rare mutations in single genes that are associated with a substantially increased schizophrenia risk have been identified (96, 97). In addition, it has been shown that there is a burden of rare variants in genes intolerant of loss-of-function variants in schizophrenia (98). It is likely that the number of identified rare mutations in single genes in schizophrenia will increase considerably in the near future, and more information will be available from the phenotypic spectrum associated with them.

A common feature in CNVs and rare mutations is an association with a variety of neurodevelopmental problems, including intellectual disability, and patients with schizophrenia who have these rare variants have worse cognitive functioning than other patients with schizophrenia (96–98). Therefore, genetic testing for these rare variants may be useful for FEP patients who have a history of neurodevelopmental problems, poor cognitive functioning, and neurological symptoms. In contrast, there is currently no evidence on whether these variants are also predictive of treatment response or the long-term outcome.

There is also evidence of specific genes that are associated with both antipsychotic treatment response and side-effect risk which differ from those associated with disease risk. Alleles in the dopamine D2 receptor and in the glutamate ionotropic receptor delta type subunit 2 (GRID2) are associated with antipsychotic treatment response (99), and several genetic variants that predispose to antipsychotic-induced weight gain have been identified (100). In addition, pharmacogenetic tests related to drug metabolism are already in clinical use (101).

### Blood-based biomarkers

Besides genetics, other potential blood-based biomarkers for psychotic disorders have been studied extensively (102), and several reviews and meta-analyses have been published (102–106). Some of the main lines of research are presented below. In general, there is much more research on whether certain biomarkers cross-sectionally separate patients from healthy controls than there is research about the possible predictive value of the biomarkers in patient treatment.

The association of a dysregulated immune response and psychosis is well-established. Several pro-inflammatory cytokines are elevated in FEP patients (107–109), including drug-naïve patients (110). The changes are similar in the cerebrospinal fluid (CSF) and blood, and they occur across severe mental disorders (109). There are also changes in the levels of distinct lymphocyte subtypes (111). While meta-analyses initially suggested that antipsychotic medication might decrease pro-inflammatory activation (108), a later meta-analysis did not find a significant medication effect (110). Further signals of a change in immune response come from associations with markers of oxidative stress (112) and the activation of the complement system (113). While various markers of immune response have been found to correlate with clinical features, such as structural brain abnormalities, symptoms and cognitive deficits (114–116), less is known about their predictive value. These biomarkers are part of a dynamic signaling network, and we currently do not fully understand their temporal patterns and variation in early psychosis. For other medical conditions, concentrations of immunological molecules in different tissues have been shown to be quite rapidly changing (117), which is understandable given their role in the coordination of immune response. In early psychosis, there may also be other factors, like sleep deprivation (118), which may contribute to the pro-inflammatory activation. The question remains open regarding to what extent inflammation might be secondary to metabolic changes, or vice versa. More information is needed on such confounding factors before inflammatory markers can be introduced as diagnostic or prognostic biomarkers.

C-reactive protein (CRP) has been the most commonly used measure of inflammation. In the largest meta-analysis on CRP levels and psychotic disorders, CRP levels were increased in both drug-naïve and unmedicated patients, as well as after the onset of psychosis (119), although one study with only drug-naïve FEP patients did not detect any difference in CRP between cases and controls (120). In Mendelian randomization studies, genetic variants leading to increased CRP levels are not associated with an increased risk of schizophrenia (121, 122), suggesting that the association between elevated CRP and schizophrenia is not caused by a common genetic mechanism. CRP is associated with increased mortality risk but not with the risk of rehospitalization in patients with depression, bipolar disorder or schizophrenia (123). However, CRP has been studied and suggested as a biomarker for numerous acute and chronic diseases, and it remains to be studied whether its best value in treating patients with psychotic disorders would actually be found in assessing the risk of comorbidities (e.g., in cardiovascular risk assessment) (124).

The anti-N-methyl-D-aspartate-type glutamate receptor (anti-NMDAR) encephalitis can in some cases present with prominent psychotic symptoms (125, 126). The identification of encephalitis in patients with early psychosis is crucial, as over 75% of patients with classic anti-NMDAR encephalitis have substantial recovery with specific treatments, while antipsychotic treatment is not effective (125). Based on several reports, however, the diagnostic evaluation of autoimmune encephalitis in FEP can be focused on those presenting specific neurological symptoms (125). Other than anti-NMDAR antibodies, autoantibodies detected in autoimmune encephalitis seem to remain negative in patients with isolated early psychotic symptoms (127). However, a recent study found that patients with schizophrenia and NMDAR antibodies suffer from more severe symptoms than other patients with schizophrenia despite a negative test for encephalitis (128). Therefore, the role of autoantibodies as biomarkers of longer-term outcomes deserves attention in future studies.

Several endocrine markers have also been studied in FEP, but it is unclear whether they reflect primary changes or secondary effects. They correlate with inflammation and metabolic changes, and the link to early trauma and stress response is strong for all of them. For instance, Misiak et al. have reviewed evidence for increased levels of testosterone and dehydroepiandrosterone in FEP (129) and suggest that these alterations might be related to a stress response. In drug-naïve FEP patients, there is evidence for an increased level of morning cortisol, cortisol awakening response, and increased prolactin levels, all of which may refer to a dysregulated hypothalamic-pituitary-adrenal (HPA) axis (130). In clinical high-risk patients, elevated cortisol predicted transitioning to psychosis (131), and in FEP patients it is correlated with the severity of symptoms and aggression (102). Its predictive value is less clear (102). Increased leptin in psychosis is mostly explained by a medication effect on weight gain, and a meta-analysis did not find significant changes in drug-naïve patients (132).

Peripheral monoamines and their metabolites have been studied as candidate biomarkers for treatment response in FEP. Elevated levels of plasma homovanillic acid, the principal dopamine metabolite, and the norepinephrine metabolite 3-methoxy-4-hydroxyphenylglycol have been associated with a better treatment response in a few, relatively small, studies (102). Tryptophan metabolite kynurenine acid (KYNA) has been studied extensively in recent years. A meta-analysis found that KYNA levels are elevated in CSF, but not in plasma, in patients with schizophrenia (133), and KYNA elevation is linked to proinflammatory activation (134). In addition, ratios of different tryptophan metabolites have predicted treatment response in patients with schizophrenia (135).

In the search for biomarkers, “omics”-based methodologies are becoming widely used. Proteomic methods have been used to identify the biomarkers that differentiate FEP or first-episode schizophrenia patients from controls (104). There tends to be consistency between studies in the identified biological pathways, many of which have already been mentioned before; the most important were the acute-phase pathway, communication between innate and adaptive immune cells, lipid and glucose metabolism, blood formation and clotting, and the stress response (104). Studies using the metabolomics and lipidomics approaches in schizophrenia research were recently reviewed by Davison et al. (106). The most consistent findings across studies have been elevated 3-methoxy-4-hydroxyphenylglycol, glutamate, lipid peroxidation metabolites, and triglycerides (triacylglycerols), and decreased creatinine, vitamins (B6, D, E, and folate), phosphatidylcholines, phosphatidylethanolamines, and polyunsaturated fatty acids (106). Several groups have suggested biomarker panels that differentiate patients with schizophrenia from healthy controls, but there is little overlap in individual metabolites in these panels (106). Fewer studies have investigated whether these biomarkers have prognostic value. As examples of such studies, 3-hydroxykynurenine was predictive of symptom improvement in first-episode schizophrenia in one study (136), and the higher baseline levels of triacylglycerols with a low carbon number and double-bond count were predictive of weight gain in FEP in another study (137). Of note is that low levels in some biomarkers of nutrition, like vitamin D, require supplementation, and it may be relevant to monitor them as a part of the general health assessment of patients with FEP.

Increasingly, various biomarkers are combined into panels in order to have better predictive value, resembling the PRS of genetic studies. Typically, individual biomarkers and their analytical methods differ between research groups, and therefore this line of work is difficult to summarize. One example is given in the following. Sabine Bahn's group developed and validated a biomarker panel using five independent study samples (138). Their panel consisted of 26 analytes measuring lipid transport, inflammation, the immune system, hormonal signaling, growth factor signaling and the clotting cascade (138). The predictive power of the panel to identify patients who later developed psychosis from two independent at-risk cohorts was good (the area under the curve 0.82–0.90) (138). In the North American Prodrome Longitudinal Study, a classifier was built that was able to predict psychosis conversion with an accuracy of 0.90 using 15 analytes measuring lipid transport, immune system, hormonal signaling and the clotting cascade (139). Of note is that, while the profile of analytes were fairly similar in these two studies, only three individual analytes (interleukin 8, thyroid stimulating hormone, and factor VII) were the same in both panels (138, 139). While this example is not about prognostic biomarkers, it illustrates the challenges in replicating this type of biomarker panels.

### Physical comorbidities and their predictors

CVDs are a leading cause of excess mortality in schizophrenia (6, 7), and preventing CVD risk factors (such as impaired glucose tolerance and diabetes, obesity and dyslipidemia) in patients with FEP is an important target.

Weight gain affects a significant proportion of individuals using antipsychotic medication and is associated with almost all antipsychotics (8, 140). However, there is considerable individual variation in antipsychotic-induced weight gain. Various risk factors for antipsychotic-induced weight gain have been reported in the literature but only with limited consistency. Several studies have found that a young age and low BMI before antipsychotic treatment predict a larger increase in weight (141–144). Other reported risk factors for weight gain include female sex, a non-white ethnic background, negative symptoms, poor social functioning, and co-medications, while smoking and cannabis use have been associated with less weight gain (141–146). A dysregulated glucose metabolism may also mark an increased risk of weight gain (147, 148). Early weight gain predicted further weight increase in a longer follow-up (149). In a meta-analysis of the genetic factors affecting antipsychotic-induced weight gain, 13 single-nucleotide polymorphisms (SNPs) in nine genes were significant predictors, with the most significant effect sizes for SNPs in ADRA2A, DRD2, HTR2C, and MC4R (100). However, a PRS computed from the 6 SNPs with the largest effect on weight gain only explained 5.6% of the variance in weight gain in two cohorts of FEP patients, showing that the predictive value of genetic markers is modest (100). Combining the genetic findings with clinical risk factors for weight gain has resulted in modest improvements when compared to only using clinical factors. Whereas, in one study genetic data (SNPs from GWA studies of BMI and candidate gene studies) increased the prediction accuracy compared to using clinical data alone (150), in another study adding data from PRSs did not improve the prediction of weight gain compared to the clinical information (148).

Regarding impaired glucose tolerance and dyslipidemias, antipsychotic medication contributes to them, but many markers of prediabetes—including insulin resistance, impaired glucose tolerance, and elevated triglycerides—are more common in drug-naïve patients with FEP than in age- and gender-matched controls (130, 151, 152). Insulin resistance seems to precede obesity in FEP (153, 154), and antipsychotic-naïve FEP patients do not differ in BMI from controls (155). Antipsychotics have a more rapid effect on insulin sensitivity than on weight, which has also been shown in healthy volunteers exposed to antipsychotics (156). Furthermore, insulin resistance predicts more increase in weight in patients with FEP during the first year of antipsychotic treatment (147). A rare, unpredictable adverse effect of several second-generation antipsychotics is type 2 diabetes manifesting as diabetic ketoacidosis (9). Similarly, there are case reports of severe triglyceridemia and acute pancreatitis related to antipsychotics (157, 158). Predictors of progression to diabetes or the risk of severe dyslipidemias in FEP are currently lacking.

The overall risk of CVDs may not be elevated in drug-naïve patients with FEP but it already increases significantly during the first 6–12 months of antipsychotic treatment (159, 160). Besides the classical risk factors, also elevated total white blood cell count and CRP levels have been associated with increased CVD risk, and increased CRP levels have been associated with mortality in psychotic disorders (123, 161). Of the other predictors of mortality, smoking increases the mortality risk due to associated diseases and medical conditions (7, 162). Antipsychotic use is associated with a lower mortality risk in several studies (23, 163), but using doses of antipsychotics that exceed the recommended dose may increase CVD mortality (164). The prediction of CVD risk for people with severe mental illness is more accurate if the traditional risk factors—smoking, diabetes, hypertension, obesity, and dyslipidemia—are complemented with information on psychiatric diagnosis, the use of antipsychotics and antidepressants, and harmful alcohol use (165). Many studies have evaluated the CVD risk for patients with psychotic disorders compared to the risk for healthy controls using traditional algorithms like the Framingham risk score (166), but it has not been studied in large, prospective cohorts whether these risk algorithms should be tailored to patients with psychotic disorders.

### Suicide risk and its predictors

Up to 90% of clinical high-risk patients report suicidal ideation, between 15 and 26% of FEP patients have made at least one suicide attempt by their first treatment contacts, and 2–11% attempt to end their lives over the first year after treatment onset (167). The risk for an attempt is highest during the month preceding treatment seeking and the first 2 months following that (10, 168). Suicide attempts in the early course of illness are characterized by methods of high lethality and include most of the suicide completions (168). Long-term follow-up studies and register studies also show that most suicides occur during the first 2 years after the onset of FEP (10, 169, 170).

The predictors of a higher suicide risk include the earlier age of onset; a history of previous suicide attempts; the severity of the symptoms of depression, anxiety, and psychosis; substance abuse; being male; a high IQ and better neurocognitive functioning; a high level of education; high socio-economic status; poor premorbid adjustment; living alone; a longer DUP; insight; and a family history of suicide (29, 167, 170, 171). Compliance with treatment has been demonstrated to reduce the suicide risk (171), whereas the highest OR for suicides has been found for patients with a previous history of suicide attempts and a history of alcohol abuse (172).

The neurobiology of suicidality was recently reviewed (173); the presented biological mechanisms have all also been of interest in the etiological research of early psychosis. Several investigators have also presented ML algorithms to identify suicidal patients in a retrospective setting. The prediction has been done based on the information from health records, either through an expert review (154) or using language analysis (155). Applications predicting the future in the predictive models could detect half of the suicide attempts and deaths during the next 60 days (156). These models were not developed specifically for FEP patients, however.

### Substance use

Continuing substance use is predictive of several adverse outcomes in FEP patients. It is associated with a higher risk of relapse and a poorer 10-year outcome, whereas patients who discontinued substance use within 2 years after the first psychotic episode had similar 10-year outcome as those who had no history of substance use (174). Sustained cannabis use in FEP patients is associated with higher relapse rates, longer hospital admissions, and more severe positive symptoms (175, 176). As reviewed above, substance abuse is a risk factor for suicidal behavior. In addition, smoking is a major risk factor for premature mortality in patients with psychosis, as it is in the general population (7, 162). To summarize, the outcome is worse across many domains in FEP patients with persistent SUD but not for those who discontinue substance use. Therefore, treating a comorbid SUD should be an integral part of treatment of FEP.

### Limitations

In order to build reliable prediction tools, large and representative patient samples are needed. Clinical follow-up studies, which require good collaboration and interest from the participants, always have some attrition. In the Oslo Schizophrenia Recovery study, 10% of those fully recovered were no longer in any contact with mental health services (38). These individuals are easily lost in follow-up, which should be taken into account when estimating the recovery rates and predictors of remission and recovery (38). On the other hand, patients with prominent disorganized symptoms may be too ill to give an informed consent in the first place. In retrospective studies where complete information has been available (e.g., from a lifetime review of medical records), about 15–20% of patients with schizophrenia have had the disorganized subtype, which is characterized by poor functioning from the onset of illness and a considerably poorer long-term outcome compared to other schizophrenia subtypes (177, 178). If these patients are underrepresented in clinical studies which have intensive protocols and require the capacity to give informed consent, this could explain why disorganized symptoms have not emerged as notable outcome predictors in many studies. Register-based studies are able to overcome selective attrition, but clinical data available in health care registers is often superficial and the information available from those who have dropped out from treatment is limited, even in countries where different types of nationwide registers exist (e.g., registers on sociodemographic factors like work and income).

A problem related to blood-based biomarkers is that psychiatric research rarely fully considers what is already known about these biomarkers in other medical fields. Many suggested biomarkers have stronger research evidence from another medical field and are affected by various confounding factors, like stress, sleep, nutrition, smoking, exercise, and BMI. There may be substantial effects of antipsychotic and other psychotropic medication on various biomarkers, and these have not yet been fully characterized. On the other hand, some biomarkers may only be relevant for a specific subtype of FEP. These factors should be carefully examined before recommending any blood-based biomarker for clinical use.

## Future directions

Methodological advancements may lead to better biomarkers from one modality or to improved prediction by an optimal combination of various markers. Clinicians also need better tools for interpretation of predictive information.

### Prediction strategies and tools

Disease risk calculators have been available in many medical fields for decades (179, 180) but are only now emerging in psychiatry. As an example, a risk calculator for predicting the psychosis conversion risk in patients with a clinical high-risk state was recently published (181). The calculator combined scores on prodromal symptom severity, decline in social functioning, and verbal learning and memory (181). However, with increasing predicted risk, the sensitivity of the test became quite modest (181).

Outcome prediction tools for FEP do not currently exist. Two recent studies have used simulated data to illustrate an approach to developing such tools. Schubert et al. illustrated how multimodal sociodemographic, clinical, psychological, imaging, and other neurobiological information could be used to develop a prediction tool for the different disease trajectories of FEP (47). Schmidt et al. (105) suggested sequential testing as another method to improve outcome testing. They presented such a model in the context of clinical high-risk research, where it was shown that sequential testing, first with clinical markers and then with different biological markers—including MRI, EEG, and blood biomarkers—was able to markedly improve the accuracy of predicting future psychosis (105). Of the patients who showed increased risk in all three tests, only 2% did not convert to psychosis (105). The challenge of sequential testing is compromised sensitivity as each known test misses true converters. ML provides promising tools for increasing sensitivity.

### Machine learning

ML refers to various tools that learn to classify or score new data once given a training data set (182). Thus, it offers a technique with which to attain a computer-assisted clinical decision-making tool. In unsupervised ML, the algorithm differentiates naturally occurring classes in the data set. In supervised ML, the ML algorithm is given a known outcome, for example a diagnosis or a level of functioning. ML algorithms can handle enormous quantities of data and find, in addition to linear associations, non-linear associations, including those between an outcome and different combinations of data features (182). The resulting complexity of the model easily leads to overfitting, that is, high accuracy in the training set, but poor generalizability to independent data sets. The goal of ML analysis is to optimize the model so that the algorithm performs optimally, both in the training set and in an independent test set (182, 183).

There are multiple ML methods available, including neural networks and support vector machine. By some analogy to the brain's functioning, neural networks use a set of hierarchical layers that correspond to different levels of abstraction (182). A support vector machine finds the largest marginal between the data points that separates the defined outcomes (184). By using a matrix of similarities between data points (the kernel), high dimensional data sets, such as brain images, can be used efficiently even in small samples (184). ML tools are not restricted to a single modality, such as a clinical data set or a brain image, and different modalities can be combined. They may have complementary information, and emerging evidence suggests multimodal methods are likely to enhance accuracy (185). Results from ML analyses in a single modality can be used as either a concatenated or separate input to a new ML model, or they can be used in sequential testing.

### Novel imaging methods

Non-invasive brain imaging methods are developing constantly. For example, an increase in MRI field strengths increases the signal-to-noise ratio and benefits translational research in FEP. New imaging methods can reveal new aspects of the brain (186). Such methods may provide complementary data that increase the accuracy of predictive models either alone or combined with other data.

In addition to the development of devices and radioligands, functional imaging may benefit from task development. Tasks are necessary in addition to resting-state imaging as they synchronize mental states and related brain functions, resulting in imaging signals that are comparable across subjects and time points. A limitation in common tasks—such as sensory, motor, or cognitive tasks—is that while they are well-controlled, they only activate a very limited set of brain circuitries. In contrast, psychotic disorders are related to multiple functional alterations across the brain (187). Therefore, predictive models would likely benefit from rich naturalistic stimuli—such as music, stories, or movies—that activate most of the brain across subjects in a synchronous manner (188, 189).

### Biomarkers

Novel methodologies may lead to new biomarker discoveries. Methodologies are developing rapidly in genetics and various fields of “omics” research. An example of a novel strategy is untargeted screening for IgG reactivity to fragments of human proteins, which has identified potentially interesting novel autoantibodies in FEP (190). However, in order to have new biomarkers for clinical use, several steps are needed after initial discovery (191). As noted by Fond et al. (102), biomarkers need to be “accurate, reproducible, acceptable to the patient, easy to interpret, and have an adequate sensitivity and specificity.” This means that the procedures for assays need to be optimized and their reproducibility within and between laboratories ensured (191). Possible important covariates affecting the biomarker level, like age or sex, need to be identified and taken into account (191). The frequency of true-positive and false-positive results must be determined in different clinical settings, after which the criteria for a positive screening test need to be defined (191). If biomarkers are combined as risk scores, this process is needed both for the individual components and the combined score (191). Finally, the cost-effectiveness of biomarkers needs to be demonstrated (191). For the great majority of biomarkers presented in this review, the critical steps regarding reproducibility and accuracy in clinical settings have not yet been accomplished.

### The validation of multimodal predictive models

To be implemented clinically, multimodal predictive models need to be validated. Discovery studies tend to be small and need to be replicated in larger samples. Multicenter studies can provide the necessary evidence that a model functions independently of certain samples and investigators. It has been shown, however, that the high heterogeneity of a sample decreases the performance of the model in multicenter studies (192). Thus, the model may need to be finally optimized in the local population. Finally, it is not enough to predict the future for those with FEP, but the predictions need to serve the patients' needs. Thus, the ultimate goal is to show that validated predictive models help to enhance the outcomes of the patients in randomized controlled settings.

No predictive model can be deterministic as the future of many internal and environmental factors is impossible to predict. Thus, to optimize predictive models, they should be updated based on follow-up data. Such updates may not need costly examinations by the health care system. Knowledge about daily experiences can be collected by mobile applications (193) and information about changes in movements and communication can be collected from mobile phones without the need of a patient actively inputting the data. Such information may help to update the multimodal prediction models of the future.

### User interface

Multimodal prediction models need visualization tools in order to be clinically useful. Naturally, before such tools are incorporated into clinical practice, there has to be robust evidence that the prediction model itself is valid. An example of a computer-assisted clinical decision-making tool is the Disease State Index (DSI) and the Disease State Fingerprint tool, which were initially developed to predict Alzheimer's disease risk in elderly people with mild cognitive impairment and they have been expanded for use in differentiating the separate types of dementias (194–196). Two important features in the DSI are that full information from all potential predictors is not needed from an individual patient to use the prediction model and also that the visualization of different risk components is easy to interpret. Furthermore, the tool illustrates whether there are inconsistencies between different predictors, in other words if some outcome predictors point out to a poor, other predictors indicate a better outcome. Such tools would be very useful especially for enhancing the use of brain imaging and cognitive data in outcome prediction for FEP.

### Conclusions

The personalized treatment of FEP will need predictive tools. At a group level, there are already many clinical parameters that predict different outcomes, but these should be transformed into an individual-level prediction, where patients typically have mixed features—some predicting a better outcome, others a worse outcome. Methodological advancements such as ML will help in developing multimodal prediction tools and in transforming the research findings into clinical practice. User-friendly interfaces are needed for such tools. The possibility to use such platforms with incomplete information is also important. At the same time, it has to be remembered that scientific breakthroughs are often unpredictable. The research field needs to ensure that novel findings, for example those emerging from genetic studies, are thoroughly investigated—it is possible that the biomarkers available within 10 years are completely outside the lists mentioned in this review.

## Author contributions

All authors listed have made a substantial, direct and intellectual contribution to the work, and approved it for publication.

### Conflict of interest statement

The authors declare that the research was conducted in the absence of any commercial or financial relationships that could be construed as a potential conflict of interest.
